# Modulation of the Main Resistance-Associated ABC Transporter’s Expression by Plant Flavonol Isorhamnetin

**DOI:** 10.3390/ph18040494

**Published:** 2025-03-28

**Authors:** Milena Milutinović, Filip Ristanović, Nikola Radenković, Danijela Cvetković, Sandra Radenković, Milan Stanković, Danijela Nikodijević

**Affiliations:** 1 Department of Biology and Ecology, Faculty of Science, University of Kragujevac, Radoja Domanovića 12, 34000 Kragujevac, Serbiamstankovic@kg.ac.rs (M.S.); 2 Faculty of Medical Science, University of Kragujevac, P.O. Box 124, 34000 Kragujevac, Serbia; 3 Institute for Oncology and Radiology of Serbia, Pasterova 14, 11000 Beograd, Serbia

**Keywords:** *BCRP*, isorhamnetin, cancer cell resistance, MDR, MRPs, P-gp

## Abstract

**Background/Objectives**: Multidrug resistance is one the leading problems in cancer treatment, where the overexpression of P-gp and other drug efflux pumps is regarded as the primary cause. With the intention to develop transporter inhibitors, natural products such as phenolics have shown great potential and diverse attention recently. Among these, isorhamnetin (ISO), an O-methylated flavonol, is predominantly found in the fruits and leaves of various plants. Thus, this study aimed to investigate the effects of ISO on the mRNA expression of membrane transporters P-gp, *BCRP*, *MRP 1*, *2*, and *5*, the protein expression of P-gp, as well as the *GSTP1* and GSH content in DLD1 and HCT-116 colon cancer cells. **Methods**: The cytotoxic effect of isorhamnetin is assessed using an MTT test, while qPCR and immunocytochemistry methods were used to determine gene and protein expression levels. The concentration of reduced glutathione was determined using the colorimetric method. **Results**: Based on the results, ISO can modulate the expression of transporters responsible for the resistance development (all transporters on the transcriptional level were downregulated in DLD1 cells, while only *MRP1* on HCT-116 cells, and reduced P-gp protein expression on both investigated cell lines). Increased glutathione content in treated cells and *GSTP1* expression suggest metabolizing the ISO and potential ejection with GSH-dependent pumps. **Conclusions**: Thus, in future experiments, ISO as a natural medicinal compound could be used as a chemosensitizer to prevent or overcome membrane transporter-mediated drug resistance.

## 1. Introduction

There is intensive research on new types of tumor therapies, and many have been developed, but chemotherapy is still the main approach for the treatment of colorectal cancer. However, in parallel with developing new therapeutic strategies, there are numerous specific problems, such as adverse side effects, toxicity at high doses unsuitable for achieving a therapeutic effect, toxicity on the normal/nonchanged cells, and the development of cancer cell resistance [1].

The multidrug resistance (MDR) of cancer treatment manifests cross-resistance to several structurally different chemo-agents, even those with various targets. There are numerous ways to adapt tumor cells to the treatment and achieve resistance, such as avoiding apoptosis, decreasing drug uptake, inactivating drugs via biotransformation and metabolism, activating epigenetic regulation, changing molecule target site for drugs, increasing drug efflux, and many others [2,3].

Among the different mechanisms of resistance development, the most common is increased drug efflux by ATP-binding cassette (ABC) transporters. More than 20 members of ABC transporters are reported to be involved in developing MDR mechanisms [4,5]. Resistance mainly occurs due to the increased expression of the *MDR1* gene (*ABCB1*) for the P-gp and/or its enhanced activity. However, other transporters also contribute to developing resistance in colorectal carcinoma cells, such as the BCRP (Breast Cancer Resistance Protein) and MRPs (Multidrug Resistance Proteins). Their overexpression, especially in colorectal cancer cells, is initially destined for the xenobiotic metabolism and elimination from epithelium [2,3]. P-glycoprotein exports chemotherapeutics from tumor cells, preventing tumor cells from reaching the concentration of chemotherapeutics necessary to manifest a therapeutic effect. Thus, the inhibition of P-gp and its effect, in competition with the chemotherapeutic agent as a substrate for P-gp transporter, may result in the reduction of the efflux of the drug from the cell and thereby the increase of its intracellular concentration, enhancing its effect [1,6,7].

Given that resistance is a significant problem in chemotherapeutics, there are increasingly more strategies for overcoming it. For this reason, various therapeutic approaches are being considered and used. Numerous studies have been conducted to reduce the expression of these transporters or block/inhibit their activity to reduce the efflux of therapeutics. Different P-gp inhibitors were tested, like substances specific to the substrate-binding site of the P-gp transporter, blocking agents for ATP necessary for the function of this pump, gene transporter inhibitors, microRNA as regulators of the *MDR1* gene expression, etc. [8]. To overcome these problems, substances of plant origin have been the subject of numerous experimental studies. Plants represent an inexhaustible source of bioactive compounds and various secondary metabolites with a wide range of properties and health benefits [9]. The main natural substances that can modulate MDR are flavonoids, stilbenes, coumarins, terpenoids, alkaloids, and saponins, which present the fourth generation of inhibitors [10].

Isorhamnetin is a flavonol present in the leaves, flowers, and fruits of *Hippophae rhamnoides* L., *Ginkgo biloba* L., and many other species. It is one of the metabolites of quercetin and is also called 3-O-methyl quercetin [11]. It has a wide range of pharmacological effects, such as anti-inflammatory, antibacterial, and antiviral properties [12,13,14], as well as human benefits for many disorders, including cardiovascular disease protection, the prevention of neurodegenerative diseases via neuroprotection and the improvement of memory and cognition, and many other [15,16,17,18,19].

Anticancer properties achieve the cross-inhibition of cancer cell proliferation, induction of apoptosis, suppression of tumors, and regulation of the protooncogene, affecting many other signal pathways [20]. The cytotoxicity of many different cancer cell lines has been reported, such as lungs, breast, and pancreas [18]. Cytotoxicity was also confirmed on colorectal cancer cells, including HCT-116, SW480, and HT-29 cell lines [21,22]. Although data in the literature show some antitumor properties, its ability to alter the expression of transporters or other resistance biomarkers has not been investigated.

Considering the need to explore the new inhibitors and due to the insufficient data regarding the potential of ISO to prevent or reverse cancer cell resistance, this study aims to investigate the effects of ISO on membrane transporters (P-gp, BCRP, MRP 1, 2, and 5), the expression on a transcriptional level, and the protein expression of P-gp in DLD1 and HCT-116 colorectal cancer cells. Then, the effects of ISO on the *GSTP1* expression and reduced glutathione (GSH) concentration in tested cells are evaluated as a mediator of the export of xenobiotics via glutathione-dependent pumps (MRP1 and MRP2).

## 2. Results

### 2.1. Cytotoxicity of ISO

**Figure 1:**
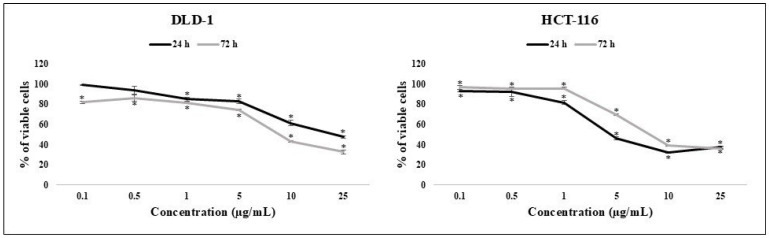
Effect of ISO on the viability of DLD-1 and HCT-116 cells. The results are presented as the mean ± standard error of three independent experiments. * Statistically significant difference (*p* < 0.05) compared to control values.

The cytotoxicity of ISO was evaluated by MTT assay on two cell lines originating from the colon—DLD1 and HCT-116. The graphs of the cell viability curves show that ISO inhibits the cell growth of colon cancer cell lines DLD1 and HCT-116 at 24 and 72 h (Figure 1). Measured IC_50_ values indicate potent cytotoxicity of ISO (Table 1). According to viability curves and IC_50_ values, it is evident that cytotoxicity was dose- and time-dependent on DLD1 cells, while HCT-116 cells recovered slightly after a longer period of exposure, and time dependence was not observed.

For further analysis and for the evaluation of the effect on resistance-associated biomarkers in colon cancer cells, the IC_25_ values of 10.48 µg/mL and 2.275 µg/mL were applied to DLD1 and HCT-116 cells, respectively.

### 2.2. Effect on mRNA Expression for Selected Membrane Transporters

**Figure 2:**
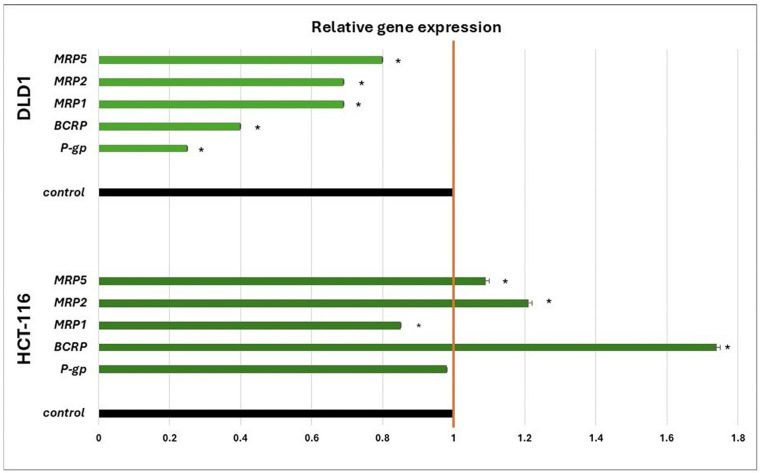
Expression of the mRNA of genes for membrane transporters in DLD1 and HCT-116 cells, under the influence of ISO (IC_25_ value) compared to the control (value 1), 24 h after treatment. * Statistically significant difference (*p* < 0.05) concerning control values.

The potential of ISO to suppress the mRNA expression of key membrane transporters (*P-gp*, *BCRP*, *MRP1*, *MRP2,* and *MRP5*) associated with cancer cell resistance occurrence was evaluated on DLD1 and HCT-116 cells. The results show that ISO significantly decreased the mRNA expression of all investigated transporters on DLD1 cells, 20 or more than that of the control (Figure 1). However, the difference in effects on both cell lines originated from the colon was observed. In HCT-116 cells, ISO significantly suppressed the mRNA expression of only *MRP1*. The expression of P-gp showed a downtrend, but it did not significantly decrease, while the expression of other transporters increased (Figure 2).

### 2.3. Effect on mRNA Expression of GSTP1

**Figure 3:**
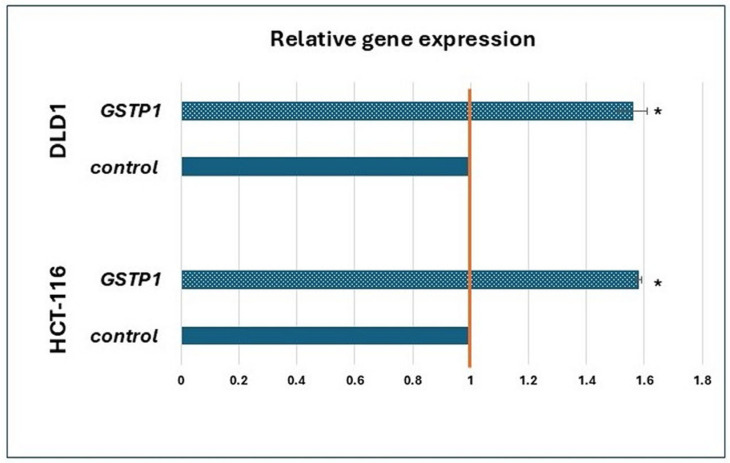
mRNA expression of the genes for *GSTP1* in DLD1 and HCT-116 cells under the influence of ISO (IC_25_ value) compared to the control (value 1), 24 h after treatment. * Statistically significant difference (*p* < 0.05) concerning control values.

The mRNA expression of *GSTP1* was increased in both the investigated cell lines, DLD1 and HCT-116, in treatment with ISO compared to the control (Figure 3).

### 2.4. Impact on GSH Concentration

**Figure 4:**
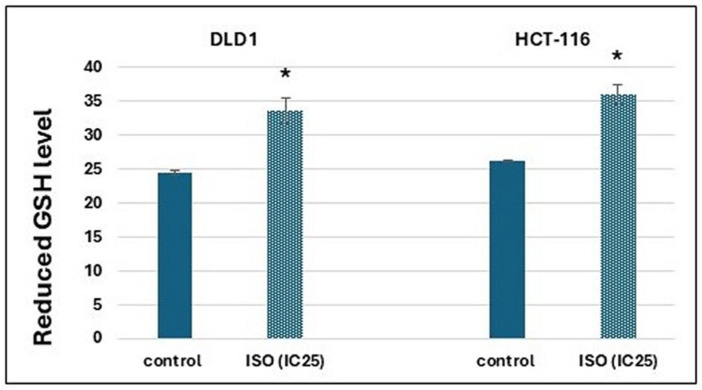
GSH concentration in DLD1 and HCT-116 cells under the influence of ISO (IC_25_), 24 h after applied treatment. The results are presented in nmol/mL and calculated per number of viable cells. * Statistically significant difference (*p* < 0.05) concerning control values.

In DLD1 and HCT-116 cells treated with ISO, the level of reduced GSH was increased compared to the non-treated control cells (Figure 4).

### 2.5. Effect on P-gp Protein Expression

**Figure 5:**
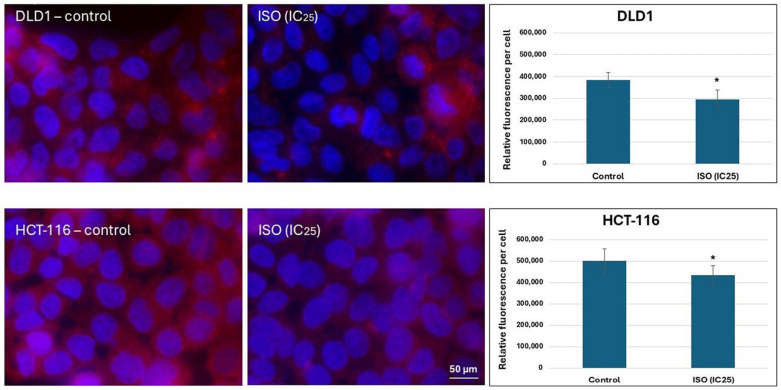
The protein level of P-gp in DLD1 and HCT-116 cells. The results are presented as the mean ± standard error of three independent experiments. * Statistically significant difference (*p* < 0.05) compared to control.

The intensity of immunofluorescence on the micrograph and the quantification of relative fluorescence (Figure 5) indicate that ISO decreased P-gp protein expression on both monitored cell lines compared to the control samples. This reduction in protein expression was more intensive in HCT-116 cells than in DLD1 cells.

## 3. Discussion

Due to multiple mutations that characterize colorectal cancer and its late detection, most patients are already at an advanced stage of the disease at the time of diagnosis. This makes this disease one of the leading causes of death worldwide [23]. Although chemotherapy, targeted therapy, and immunotherapy have shown significant success in cancer treatment, there is an increasing amount of cases of patients who are initially resistant to a particular therapy or who develop resistance during therapy [1]. Compared to other malignancies, resistance is particularly characteristic of colorectal cancers; thus, finding a combination of drugs that would overcome resistance is important.

**Figure 6:**
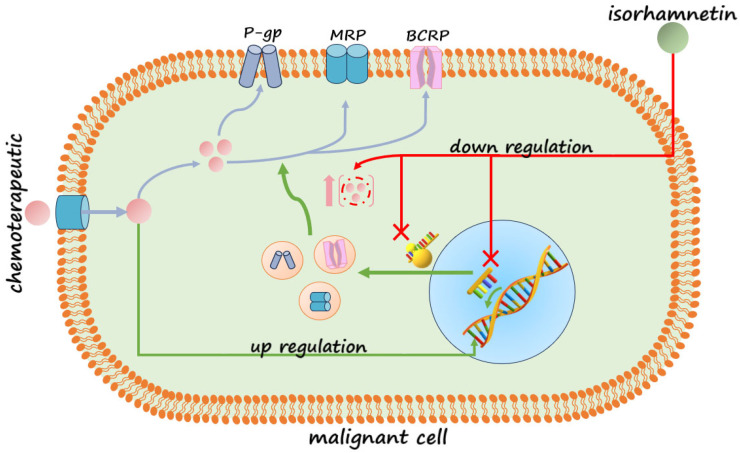
Potential mechanism of the combined therapy of ISO and the appropriate chemotherapeutic agent in a malignant cell; red arrow transporter expression inhibition pathway, green arrow transporter expression induction pathway, and blue arrow chemotherapeutics pathway.

Many different mechanisms are included in developing resistance in colorectal cancer cells, such as aberrant metabolism, transport, DNA reparation, or drug target sites [24]. However, the most common cause is the increased expression of ABC transporters, whereby the effect of chemotherapeutic agents such as 5-fluorouracil, irinotecan, and/or oxaliplatin is significantly reduced or reversed [25]. Accordingly, different approaches to solving this problem have been developed, such as combining chemotherapeutics, using transporter activity inhibitors, suppressing transporter expression by different modulators, etc. [7]. It is noticeable that, through four generations of these inhibitors, the most successful effects have been exhibited by plant secondary metabolites, such as flavonoids, phenolic acids, coumarins, alkaloids, etc. [9]. As one of the secondary metabolites, isorhamnetin is found in the fruits and leaves of various plants, including those that are medicinally important. It is also present in food products such as almonds, the onion family, grape wine, etc. Thus, as a phytochemical, it is widely available. Current research mainly focuses on the extraction of isorhamnetin from *H. rhamnoides* and *G. biloba,* which is one of the most important active ingredients [17,18]. Based on the structural and functional similarities between isorhamnetin and quercetin, which is a successful modulator of resistance, as well as a variety of its reported pharmacological applications, this study aimed to investigate the potential of ISO to reduce the expression of transporters responsible for the emergence of resistance in the human colorectal cancer cell lines DLD-1 and HCT-116. The observed results showed that the ISO treatment induced a decrease in mRNA levels for all investigated transporters and the protein expression of P-gp in DLD-1 cells. This suggests the ability of ISO to modulate the gene expression of transporters, which are most often overexpressed and associated with the development of resistance in colorectal cancer cells, and the potential to combine treatments with chemotherapeutics, which are eliminated from resistant tumor cells through these transporters. The combination therapy model is shown in Figure 6. Contrary to the reduced expression in DLD-1, in HCT-116 cells, the ISO treatment had a different trend. Namely, there was a statistically significant decrease in the level of mRNA only for the *MRP1* transporter. In contrast, the mRNA expression of *BCRP*, *MRP2*, and *MRP5* transporters increased compared to the control, and so did the protein expression of P-gp. ISO showed a decreasing trend, but a significant decrease in the expression of *P-gp* at the transcriptional level was not observed in HCT-116. An increased expression of some transporters in the HCT-116 cells treated by ISO may indicate its ejection by these transporters outside of the cells. Regardless of the origin of these cell lines from the same organ, different possibilities for developing or preventing resistance were observed.

The important result is the protein expression of P-gp, which was reduced (immunofluorescence quantification showed a statistically significant decrease) in both investigated cell lines. In addition to affecting the regulation of gene expression at the transcriptional level, ISO can potentially affect the posttranscriptional or translational level in HCT-116 cells after treatment. Other authors have reported that, among flavonoids, quercetin, whose immediate metabolite is ISO, has great potential in overcoming cancer cell resistance and inhibiting ABC transporters [26,27]. Zhou et al. [28] experimentally demonstrated that quercetin enhances the cytotoxic and proapoptotic effects of doxorubicin on SW620/Ad300 cells with an overexpression of P-gp, suggesting its inhibitory potential towards the expression of this transporter in the examined cells. Research into new substances originating from nature that can inhibit the P-gp expression and/or activity is increasingly common [10,29]. Therefore, reduced P-gp expression at both the transcriptional and protein levels in both colorectal cancer cells represents a significant result.

Previous studies showed that the PI3K/AKT pathway enhanced the expression of ABC transporters, including P-gp, MRP1, and BCRP, whose activation may reduce the response to anticancer drugs and enhance the drug efflux [30,31]. This enhanced drug efflux may be responsible for reducing ABC transporter expression, considering that other authors have reported the inhibition of the PI3K Akt mTOR pathway by ISO [32]. It was also shown that the AKT pathway may control ABC transporter expressions as part of an adaptative response to oxidative stress, which can be mitigated using quercetin-like flavonoids [33].

**Figure 7:**
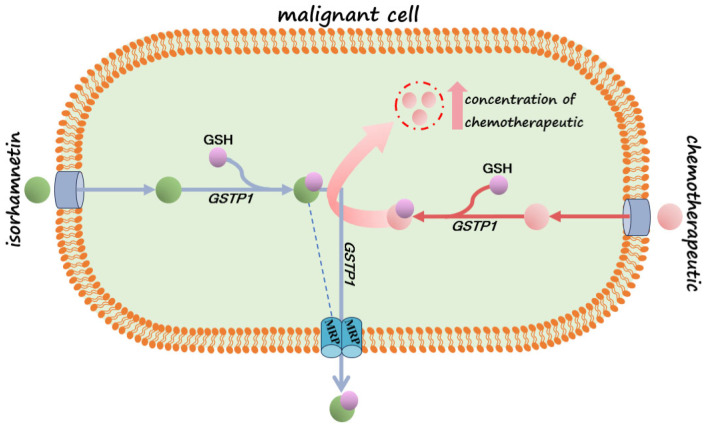
The potential of ISO as an antagonist in combined therapy with chemotherapeutics; red arrow chemotherapeutic pathway, blue arrow isorhamnetin pathway.

In addition to measuring the gene expression of membrane transporters, the level of mRNA expression for the GSTP1 enzyme, which participates in the second phase of biotransformation (xenobiotic metabolism), was also measured. It enables the conjugation of GSH with the xenobiotic for conjugate export via glutathione-dependent pumps (MRP1 and MRP2) [34]. The results show an increased mRNA level for this enzyme in both cell lines after treatment. Additionally, the increased concentration of reduced GSH in both cell lines after treatment with ISO was also observed, suggesting that cancer cells recognized ISO as a xenobiotic that needs to be thrown out. Based on this, ISO could be used in combination with chemotherapeutics as an antagonist if it shows a greater affinity for binding. In HCT-116 cells, an increased expression of GSTP1 and the MRP2 transporter, as a member of glutathione-dependent pumps, on the transcriptional level and the concentration of reduced GSH was observed in ISO treatment. This result may indicate that, in these cells, ISO is metabolized, conjugated with GSH, and thrown out of the cell. Considering that glutathione content was increased in treated cells compared to the control, as well as GSTP1 on transcriptional level results, potentially suggests to metabolize ISO through phase II of biotransformation in the cells and ejection with GSH-dependent pumps. This result can be valuable for the potential application of this flavonoid with appropriate chemotherapeutics that are similarly metabolized, considering that they would compete for the binding sites. Therefore, weaker removal of chemotherapeutics from the cells was expected. Potential combination effects are presented in Figure 7. In DLD-1 cells, it is unclear whether ISO is transported out since the expression of all GSH-dependent transporters was decreased without information about their activity.

Besides the ability of ISO to modulate the expression of resistance biomarkers, this study also confirms its noticeable cytotoxicity, presented by IC_50_ values (Table 1). Other authors reported the inhibition of tumor cell growth for colorectal, lung, breast, and skin cancer cells [35,36,37,38,39]. The recovery of HCT-116 cells after a longer treatment (higher IC_50_ value compared to 24 h) corresponds to the results of transporter expression in these cells. Increased expression of the transporters may suggest that some amount of ISO is probably ejected from the cells, which decreases its effective concentration and probably leads to less cytotoxicity after a more extended period.

Based on the results obtained, ISO has a reasonable basis for examining it in more detail with the aim of eventual application. It can be potentially used to prevent resistance development in cancer cells and/or to overcome acquired resistance in combined treatment with appropriate chemotherapeutics. The “Down” regulation of the main biomarkers of resistance indicates that ISO acts as an inhibitor of ABC transporter gene expression, where the effects on transporter activity should be investigated subsequently, while “up” regulation suggests that the binding domain of the transporter recognizes it. In the case of “Up” regulation, it is necessary to examine its binding affinity for a specific transporter in comparison to some of the chemotherapeutics used in the therapy of colorectal cancer. Competition for the same binding site would position this compound as a potential antagonist.

The observed results may contribute to the development of the fourth generation of MDR inhibitors, but more research is needed to approach and recommend clinical testing and the use of ISO to prevent or overcome membrane transporter-mediated drug resistance. A combination of in vitro, in vivo, and in silico approaches can yield significant insights into the development of MDR inhibitors, identify potential candidates for further pharmacological and clinical evaluation, and advance ongoing efforts to discover effective therapeutic agents. Recent advances in drug design and characterization, particularly molecular docking analysis, are widely applied to assess the biological potential of various compounds based on their structure [40]. Depending on the substance class and target molecule, assessing the compatibility between an active substance’s substructure and its target molecule is crucial for predicting binding affinity, interactions, inhibition, or other effects. In this context, the *in silico* analysis of the molecular interactions between ISO and ABC transporter binding sites could further elucidate their inhibitory potential. Additionally, effects on resistant cell lines should be conducted, and pharmacokinetic properties of ISO, such as oral and intestinal bioavailability and their toxic potential, should be tested. The above-mentioned investigations and predictions will enhance our understanding of the broader effects of ISO and future challenges for researchers.

## 4. Materials and Methods

### 4.1. Chemicals

Dulbecco’s Modified Eagle Medium (DMEM) and penicillin/streptomycin were bought from Capricorn Scientific GmbH, Ebsdorfergrund, Germany. Fetal bovine serum (FBS) was obtained from PAN Biotech, Aidenbach, Germany. Dimethyl sulfoxide (DMSO) and 3-[4,5-dimethylthiazol-2-yl]-2,5-diphenyltetrazolium bromide (MTT) were obtained from SERVA, Heidelberg, Germany. RNA Extracol, the reverse transcription kit (NG dART RT kit), and the qPCR Master Mix (SG/ROX qPCR Master Mix) were purchased from EURx, Gdańsk, Poland. Primers were synthesized by Microsynth, Switzerland. Diamidino-2-phenylindole (DAPI), a secondary antibody conjugated with Cy3, and the primary antibody P-gp were bought from Thermo Fisher Scientific, Waltham, USA.

Isorhamnetin (ISO)—obtained from TCI Europe N.V., Zwijndrecht, Belgium (Cat. No. 480-19-3)—was initially dissolved in DMSO and then diluted in DMEM to the concentration of ISO of 500 μg/mL and <1% of DMSO in the final stock solution (0.05% of DMSO at the highest applied concentration in MTT assay).

### 4.2. Cell Lines

Human colorectal adenocarcinoma cell lines DLD1 and HCT-116 were obtained from the American Type Culture Collection, Manassas, VA, USA. Cells were maintained in DMEM containing 10% FBS, 100 U/mL penicillin, and 100 μg/mL streptomycin at 37 °C in a 5% CO_2_ incubator (Medline, Rotherham, UK).

### 4.3. MTT Test

The cytotoxicity of ISO was determined by the MTT test [41]. The DLD1 and HCT-116 cells were seeded in 96-well plates at a density of 10^4^ cells per well. After overnight incubation, cells were treated with ISO in the concentration range of 0.1–25 μg/mL, and an assay was performed after 24 h of incubation of cells with the treatment. Nikodijević and colleagues described the experimental procedure in detail [42]. The concentration that inhibited 50% of cell growth (IC_50_) was calculated by the CalcuSyn program and used for further experiments.

### 4.4. mRNA Expression of Selected Genes

For the mRNA expression of selected genes, the total RNA was first isolated using the phenol–chloroform method [43]. The DLD1 and HCT-116 cells were seeded in 25 cm^2^ culture flasks (10^6^ cells/flask) and treated with ISO in IC_25_ concentration at 80% confluence. In control flasks, only DMEM was replaced. The isolation was performed 24 h after cell incubation with the treatment, and RNA concentrations in the samples were measured using the Eppendorf BioPhotometer Plus, Hamburg, Germany. RNA was used in the final concentration of 1 μg/μL.

The commercial NG dART RT kit was used to transcribe RNA into complementary DNA (cDNA) in the Eppendorf Mastercycler PCR, Hamburg, Germany, following the manufacturer’s procedure.

Relative mRNA quantification of the analyzed genes (*P-gp*, *BCRP*, *MRP 1*, *2*, and *5*) was performed by the SG/ROX qPCR Master Mix, using the Applied Biosystems, Quantitative Real-Time system (Applied Biosystems 7500, Real-Time PCR Software v2.0, Thermo Fisher Scientific, Waltham, MA, USA) using the recommended procedure and thermal cycling conditions. The mRNA was normalized using the β-actin as an unaltered control gene. The obtained results were analyzed by the 2^(–ΔΔCt)^ method [44]. Primer sequences for each gene are presented below:

*β-actin*

  F: AAGCAGGAGTATGACGAGTCCG;

  R: GCCTTCATACATCTCAAGTTGG.

*P-gp*

  F: GCCTGGCAGCTGGAAGACAAATACACAAAATT;

  R: CAGACAGCAGCTGACAGTCCAAGAACAGGACT.

*MRP-5*

  F: ATTTGGACCCCTTCAACCAGTAC;

  R: GGTAGCTGAGCAATACATTCTTTCAT.

*MRP-2*

  F: GCCAACTTGTGGCTGTGATAGG;

  R: ATCCAGGACTGCTGTGGGACAT.

*MRP-1*

  F: ACCCTAATCCCTGCCCAGAG;

  R: CGCATTCCTTCTTCCAGTTC.

*BCRP*

  F: TATAGCTCAGATCATTGTCACAGTC;

  R: GTTGGTCGTCAGGAAGAAGAG.

*GSTP1*

  F: TCAAAGCCTCCTGCCTATAC;

  R: AGGTGCGCAGGATGGTATT.

### 4.5. Immunofluorescence

The protein expression of P-gp was determined using immunostaining on a fluorescent microscope [45]. The DLD1 and HCT-116 cells (5 × 10^5^ cells/well) were seeded in 6-well plates on glass coverslips. At 80% of cell confluence, the medium was removed, and the cells were treated with ISO at an IC_25_ concentration. In the control cells, DMEM was replaced. The fluorescence staining procedures, as described by Nikodijević et al. [42], were performed 24 h after cell incubation with the treatment. Micrographs were made on an inverted fluorescent microscope (Nikon Ti-Eclipse, Nikon, Düsseldorf, Germany) at 600× magnification. The ImageJ software (Wayne Rasband, ImageJ, V1.54k, https://imagej.net/ij/, 15 September 2024) was used for cell fluorescence quantification, where the results were presented as relative fluorescence per cell.

### 4.6. Level of Reduced GSH

Reduced GSH concentration was performed using the colorimetric method [46]. The DLD1 and HCT-116 cells were seeded in 96-well plates at a density of 5 × 10^4^ cells per well. After overnight incubation, cells were treated with ISO in the concentration of IC_25_ μg/mL (observed by MTT assay), and an assay was performed after 24 h of incubation of cells with the treatment. Untreated cells served as the control. The experimental details were previously described by Nikodijević and colleagues [42]. The results were first expressed in nmol/mL, concerning a standard curve formed by known molar concentrations of BSA and then calculated concerning the number of viable cells (according to the results of the MTT assay).

### 4.7. Statistical Analysis

The data are expressed as the means of values from two individual experiments in triplicate for each dose from ± standard error (SE). The SPSS statistical software package (IBM SPSS Statistics Version 23, 2015) was used for statistical analysis (independent *t*-test and one-way ANOVA test). Statistical significance was set at *p* < 0.05.

## 5. Conclusions

Plant-derived phytochemicals, particularly in the fourth generation of inhibitors, hold great promise for overcoming MDR, making this research timely and crucial. There is an urgent need to identify new, safer inhibitors targeting P-glycoprotein (P-gp) and other ABC transporters, which are commonly implicated in cancer cell resistance. Our results deal with data about the observed potential of isorhamnetin, a plant-derived flavonol, to modulate ABC transporter expression on the transcriptional level and suggest its potential as an inhibitor or antagonist of membrane transporters. Among different transporters, reduced P-gp expression at the transcriptional and protein level in HCT-116 and DLD1 colorectal cancer cells represents a significant result, considering that P-gp is the most associated with the development of MDR. Additionally, our results highlight the importance of plant-derived ISO for further detailed study, where much of its potential has yet to be explored for medicinal uses and clinical application.

## Figures and Tables

**Table 1:** The cytotoxic effect of isorhamnetin expressed as IC_50_ value (µg/mL) on HCT-116 and DLD-1 cells.

Cell Line	24 h	72 h
DLD-1	20.96 ± 0.15	8.01 ± 0.67
HCT-116	4.55 ± 0.31	7.82 ± 0.09

The results are presented as the mean ± standard error of three independent experiments.

## Data Availability

Data are available upon request.
